# Supplementary material for: Conditional chemoconnectomics (cCCTomics) as a strategy for efficient and conditional targeting of chemical transmission
Source: eLife. 2024 Apr 30;12:RP91927. doi: 10.7554/eLife.91927 (PMC11060718; doi:10.7554/eLife.91927)
Supplement: Figure 3—source data 3. [file elife-91927-fig3-data3.zip › Figure 3 source data3 part 2.zip!/Figure 3-Source data 3-part2/1053f3-1-CRISPResso_Report_t6djfp6u.zip!/CRISPResso_on_1053f3_2/1c.Alignment_barplot.pdf]

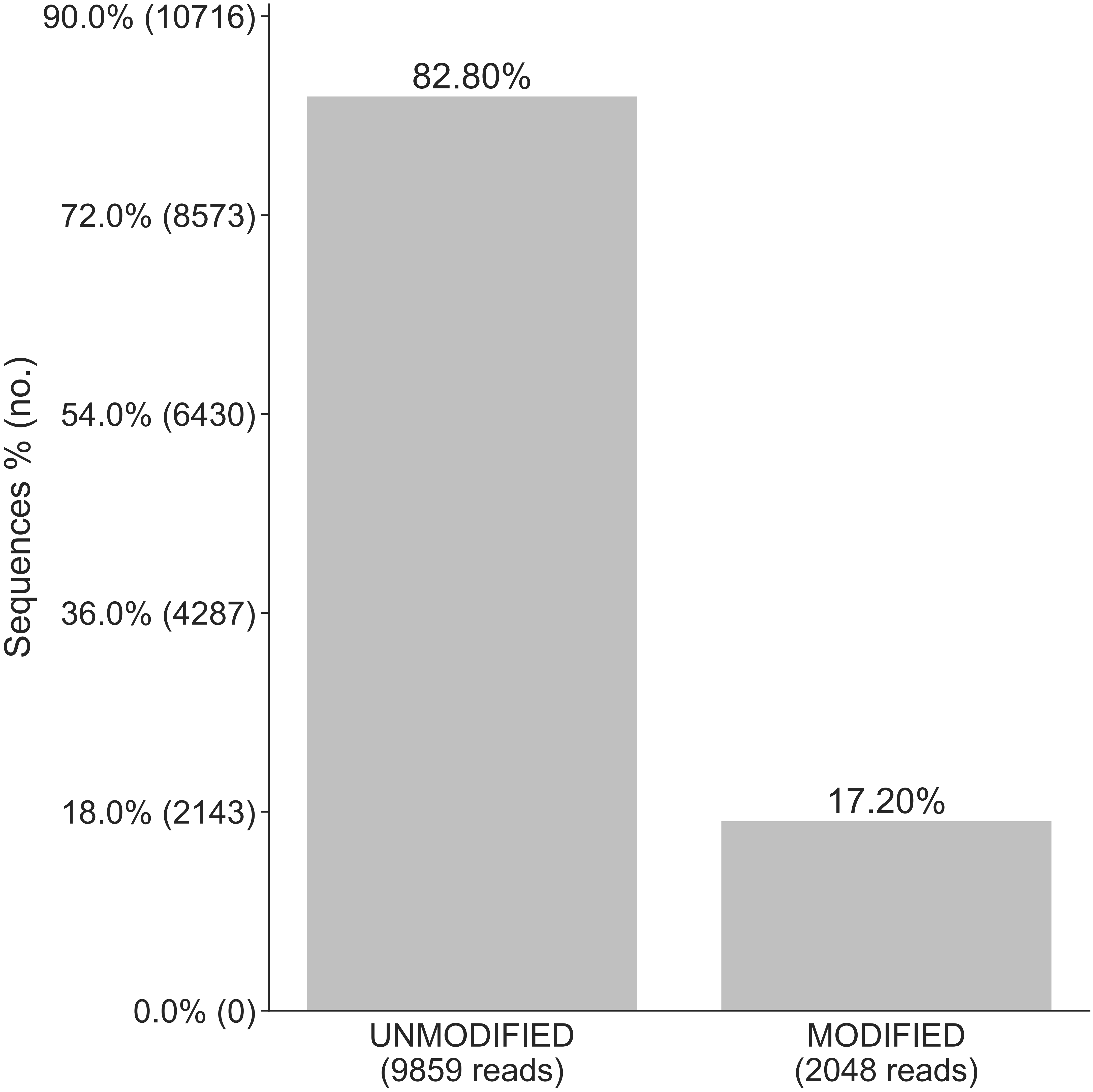

90.0% (10716)
72.0% (8573)
54.0% (6430)
36.0% (4287)
18.0% (2143)
0.0% (0)
Sequences % (no.)
82.80%
17.20%
UNMODIFIED
(9859 reads)
MODIFIED
(2048 reads)
